# Supplementary figures and images for: Clinical characteristics and prognosis differences between isolated right and left ventricular myocardial infarction in the Chinese population: a retrospective study
Source: PeerJ. 2023 Feb 28;11:e14959. doi: 10.7717/peerj.14959 (PMC9983429; doi:10.7717/peerj.14959)

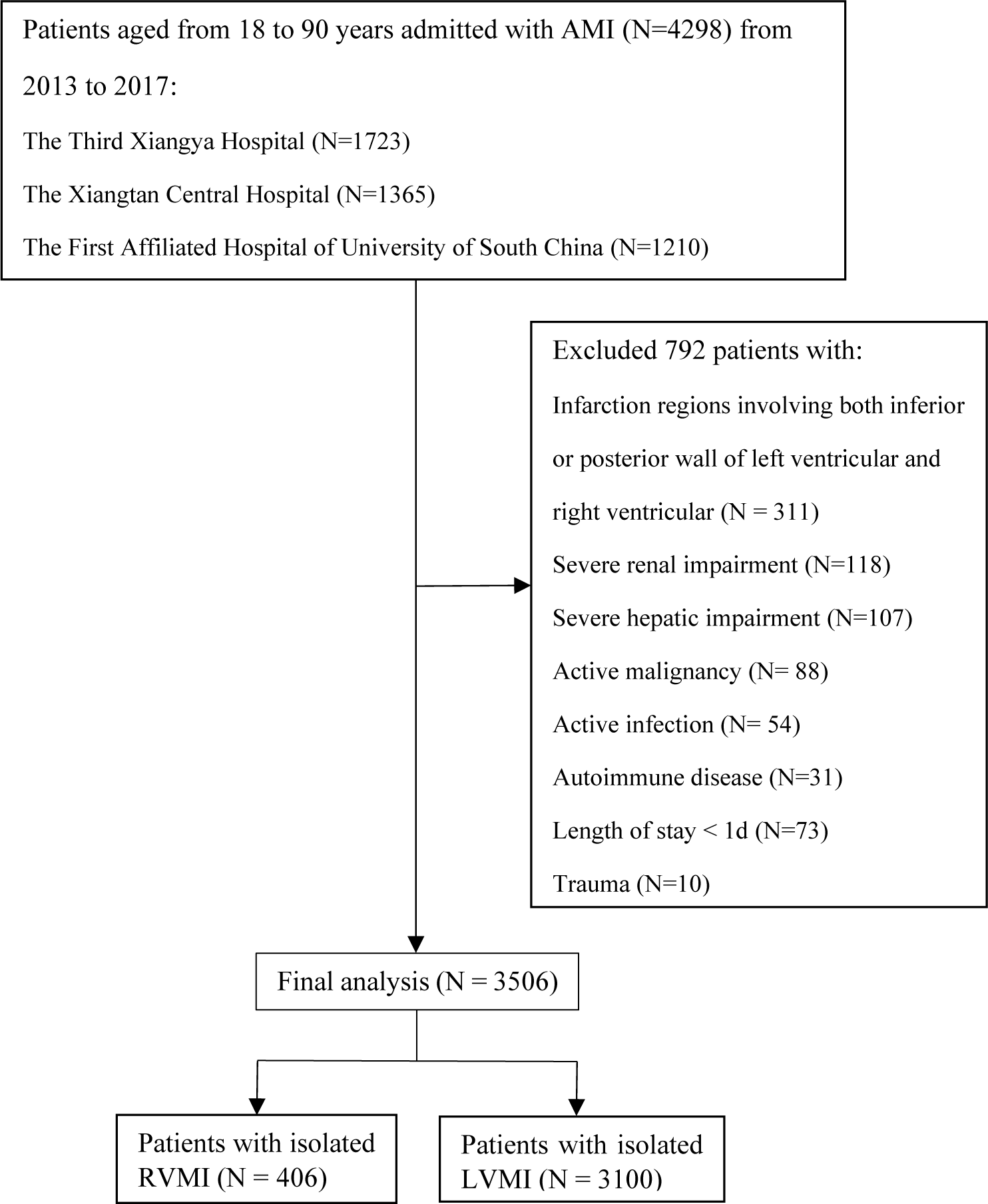

Supplement: Supplemental Information 1 — Abbreviations: AMI, acute myocardial infarction; RVMI, right ventricular myocardial infarction; LVMI, left ventricular myocardial infarction. [file peerj-11-14959-s001.png]

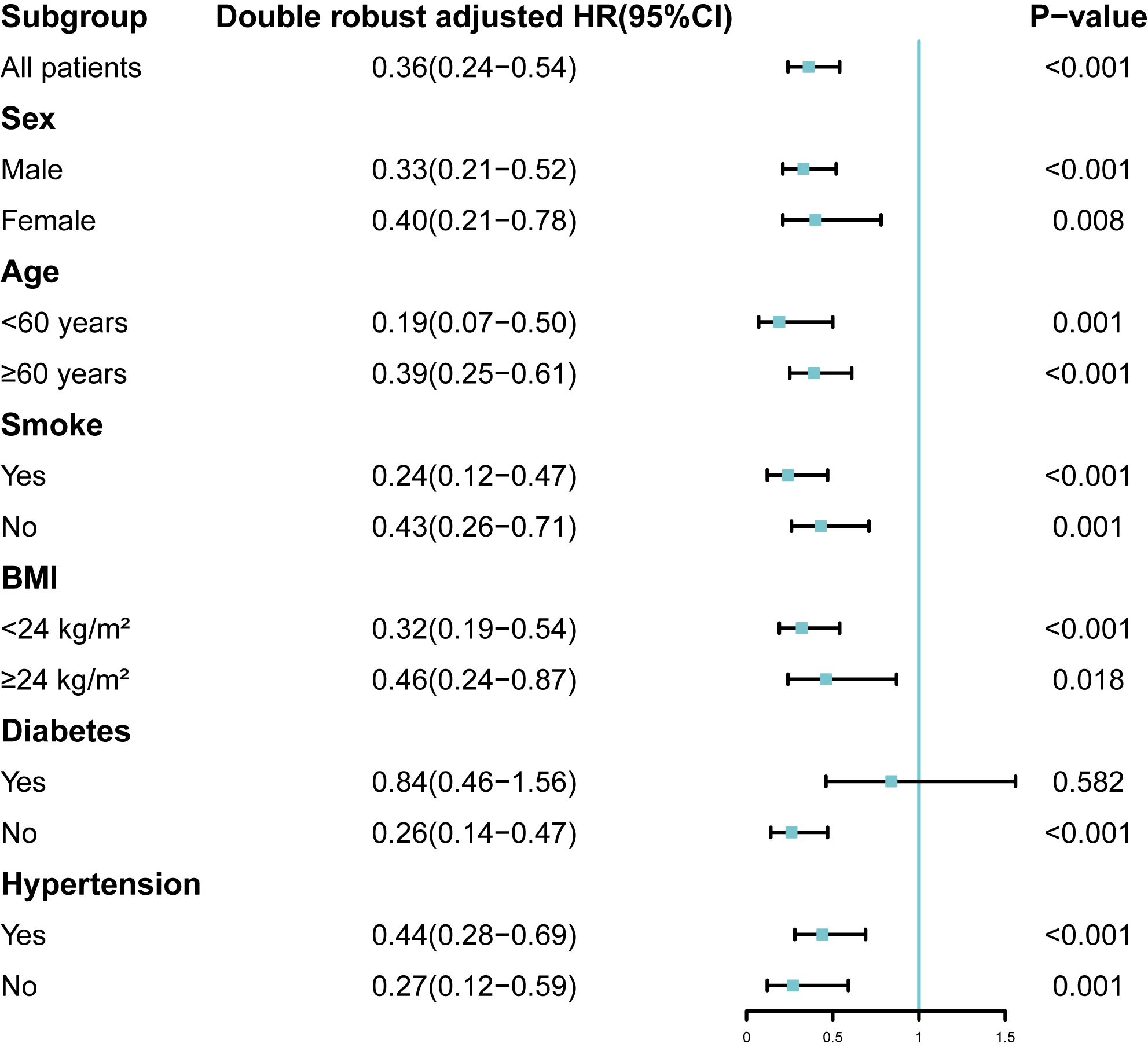

Supplement: Supplemental Information 2 — Double robust adjusted HRs and 95% CIs for left vs right ventricular myocardial infarction by specific baseline characteristics. The double robust model additionally adjusted confounders with SMD ≥0.05 after IPTW adjustment, which was age (except for age specific subgroup analysis), health insurance, clopidogrel, Killip classification, STEMI or NSTEMI, HR, SBP, DBP, FBG, LDL-C, chronic heart failure and multivessel lesion and status of revascularization. [file peerj-11-14959-s002.png]

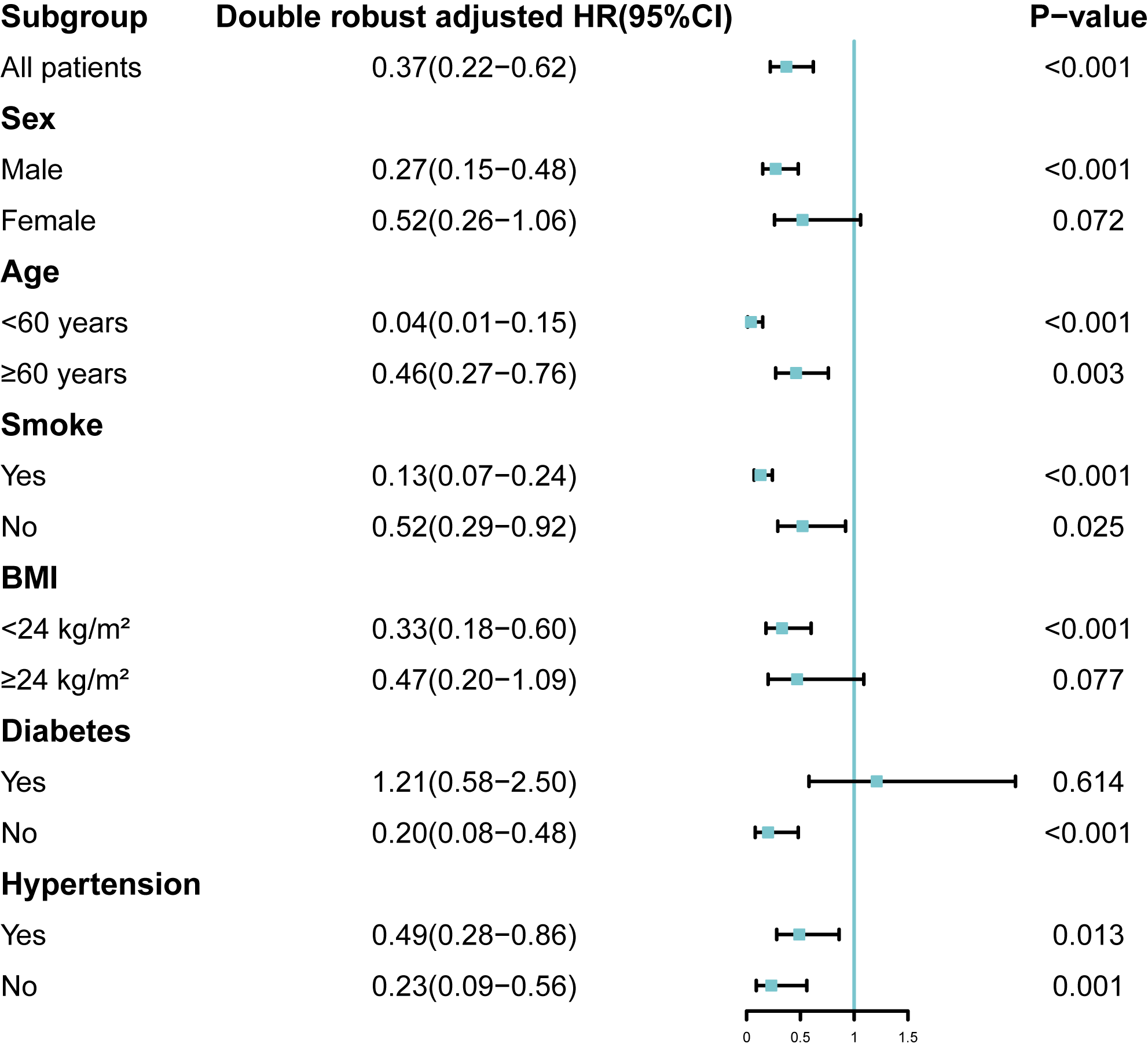

Supplement: Supplemental Information 3 — Double robust adjusted HRs and 95% CIs for left vs right ventricular myocardial infarction by specific baseline characteristics. The double robust model additionally adjusted confounders with SMD ≥0.05 after IPTW adjustment, which was age (except for age specific subgroup analysis), health insurance, clopidogrel, Killip classification, STEMI or NSTEMI, HR, SBP, DBP, FBG, LDL-C, chronic heart failure and multivessel lesion and status of revascularization. [file peerj-11-14959-s003.png]
