# Supplementary material for: Clinical characteristics and prognosis differences between isolated right and left ventricular myocardial infarction in the Chinese population: a retrospective study
Source: PeerJ. 2023 Feb 28;11:e14959. doi: 10.7717/peerj.14959 (PMC9983429; doi:10.7717/peerj.14959)
Supplement: Supplemental Information 4 — Abbreviation: ACEIs or ARBs, angiotensin-converting enzyme inhibitors or angiotensin receptor blockers; CCBs, calcium channel blockers; COPD, chronic obstructive pulmonary disease; STEMI, ST-segment elevation myocardial infarction; NSTEMI, Non-ST-segment elevation myocardial infarction; HR, heart rate; SBP, systolic blood pressure; DBP, diastolic blood pressure; FBG, fast blood glucose; LDL-C, serum low density lipoprotein-cholesterol; LVEF, left ventricular ejection fraction. [file peerj-11-14959-s004.docx]

**Supplementary Table 1. The details for each covariable in the multivariable model.**

| Covariables | Evidence |
| --- | --- |
| Age | *Bae EH et al., 2012； Gao F et al., 2015* |
| Sex | *Herlitz J et al., 2008； Gao F et al., 2015* |
| BMI | *Rana JS et al., 2004; Nigam A et al., 2005; Herlitz J et al., 2008;* |
| Smoking status | *Rodu B et al., 2012; Haig C et al., 2018* |
| ACEIs or ARBs | *Gouya G et al., 2007; Zhao X et al., 2022* |
| Beta-blockers | *Gouya G et al., 2007; Bae EH et al., 2012* |
| CCBs | *Koenig W et al., 1996* |
| Statins | *Gouya G et al., 2007; Bae EH et al., 2012* |
| Aspirin | *Gouya G et al., 2007* |
| Clopidogrel | *Gouya G et al., 2007* |
| Intravenous nitrates | *Yusuf S et al., 1988* |
| Inotropes | *Thiele H et al., 2019* |
| Diabetes | *Gao F et al., 2015; Colombo MG et al., 2015; Patel PA et al., 2015; Hall M et al., 2018* |
| Hypertension | *Kenchaiah Set al., 2004; Hall M et al., 2018* |
| Stroke | *O'Connor CM et al., 1990; Behar S et al., 1991; Hall M et al., 2018* |
| Chronic heart failure | *Abrahamsson P et al., 2009; Hall M et al., 2018* |
| COPD or asthma | *Hawkins NM et al., 2009; Hall M et al., 2018* |
| STEMI or NSTEMI | *Darling CE et al., 2013* |
| Cardiogenic shock | *Samsky MD et al., 2021* |
| Multivessel lesion | *Schulman SP et al., 1988; Mancini GB er al., 2013* |
| HR | *Henning H et al., 1979; Henning R et al., 1981* |
| SBP | *Böhm M et al., 2018* |
| DBP | *Böhm M et al., 2018* |
| FBG | *Upur H et al., 2022* |
| LDL-C | *Willerson JT et al., 1996; Cannon CP et al., 2004* |
| LVEF | *Schulman SP et al., 1988; Mancini GB er al., 2013* |
| Killip classification | *Gouya G et al., 2007* |
| Revascularization | *Gouya G et al., 2007* |

**Abbreviation**: ACEIs or ARBs, angiotensin-converting enzyme inhibitors or angiotensin receptor blockers; CCBs, calcium channel blockers; COPD, chronic obstructive pulmonary disease; STEMI, ST-segment elevation myocardial infarction; NSTEMI, Non-ST-segment elevation myocardial infarction; HR, heart rate; SBP, systolic blood pressure; DBP, diastolic blood pressure; FBG, fast blood glucose; LDL-C, serum low density lipoprotein-cholesterol; LVEF, left ventricular ejection fraction.
