# Supplementary material for: Clinical characteristics and prognosis differences between isolated right and left ventricular myocardial infarction in the Chinese population: a retrospective study
Source: PeerJ. 2023 Feb 28;11:e14959. doi: 10.7717/peerj.14959 (PMC9983429; doi:10.7717/peerj.14959)
Supplement: Supplemental Information 5 — Abbreviation: IPTW, inverse probability of treatment weighting; PSM, propensity score matching; SMD, standard mean difference; IQR, inter quartile range; BMI, body mass index; UEBMI, urban employee basic medical insurance; URBMI, urban resident basic medical insurance; NRCMS, new rural cooperative medical scheme; ACEIs or ARBs, angiotensin-converting enzyme inhibitors or angiotensin receptor blockers; CCBs, calcium channel blockers; STEMI, ST-elevation myocardial infarction; NSTEMI, Non-ST-segment elevation myocardial infarction; HR, heart rate; SBP, systolic blood pressure; DBP, diastolic blood pressure; FBG, fast blood glucose; LDL-C, serum low density lipoprotein-cholesterol; COPD, chronic obstructive pulmonary disease. 1 Adjusted for confounders including age, sex, health insurance, BMI, smoking status, in-hospital medications (ACEIs or ARBs, CCBs, beta-blockers, statins, aspirin, clopidogrel, intravenous nitrates and inotropes), medical history (diabetes, hypertension, stroke, chronic heart failure and COPD or asthma), STEMI or NSTEMI, cardiogenic shock, multivessel lesion, vital parameters and laboratory findings on admission (HR, SBP, DBP, FBG and LDL-C), LVEF, Killip classification and status of revascularization. 2 Matched for confounders including age, sex, health insurance, BMI, smoking status, in-hospital medications (ACEIs or ARBs, CCBs, beta-blockers, statins, aspirin, clopidogrel, intravenous nitrates and inotropes), medical history (diabetes, hypertension, stroke, chronic heart failure and COPD or asthma), STEMI or NSTEMI, cardiogenic shock, multivessel lesion, vital parameters and laboratory findings on admission (HR, SBP, DBP, FBG and LDL-C), LVEF, Killip classification and status of revascularization. [file peerj-11-14959-s005.docx]

**Supplementary Table 2. Baseline characteristics for patients with right and left ventricular myocardial infarction after IPTW versus PSM adjustment.**

| **Variables** | **IPTW adjustment ^1^** | | | | | **PSM adjustment ^2^** | | | | |
| --- | --- | --- | --- | --- | --- | --- | --- | --- | --- | --- |
|  | **All patients (N=6963.501)** | **Left ventricular infarction (N=3506.954)** | **Right ventricular infarction (N=3456.547)** | **P-value** | **SMD** | **All patients (N=788)** | **Left ventricular infarction (N=394)** | **Right ventricular infarction (N=394)** | **P-value** | **SMD** |
| Age, year (median [IQR]) | 65(57-73) | 64(55-72) | 65(57-73) | 0.244 | 0.087 | 65(56-72) | 65(55-72) | 65(57-73) | 0.924 | 0.002 |
| Sex, n (%) |  |  |  |  |  |  |  |  |  |  |
| Male | 1929.359(27.707) | 944.589(26.935) | 984.770(28.490) | 0.641 | 0.035 | 190(24.1) | 95(24.1) | 95(24.1) | 1.000 | <0.001 |
| Female | 5034.142(72.293) | 2562.365(73.065) | 2471.777(71.510) |  |  | 598(75.9) | 299(75.9) | 299(75.9) |  |  |
| BMI, kg/m^2^ (median [IQR]) | 23.471(22.614-24.845) | 23.580(22.676-24.840) | 23.432(22.537-24.846) | 0.629 | 0.001 |  |  |  |  |  |
| Smoke, n (%) | 3369.202(48.384) | 1684.193(48.024) | 1685.010(48.748) | 0.838 | 0.015 | 421(53.4) | 222(56.3) | 199(50.5) | 0.116 | 0.117 |
| Health insurance, n (%) | |  |  | 0.865 | 0.081 |  |  |  | 0.876 | 0.078 |
| None | 785.912(11.286) | 380.033(10.837) | 405.878(11.742) |  |  | 88(11.2) | 48(12.2) | 40(10.2) |  |  |
| URBMI | 2565.919(36.848) | 1334.289(38.047) | 1231.629(35.632) |  |  | 319(40.5) | 161(40.9) | 158(40.1) |  |  |
| UEBMI | 1228.998(17.649) | 641.603(18.295) | 587.395(16.994) |  |  | 124(15.7) | 60(15.2) | 64(16.2) |  |  |
| NRCMS | 2208.586(31.717) | 1075.136(30.657) | 1133.449(32.791) |  |  | 240(30.5) | 116(29.4) | 124(31.5) |  |  |
| Others | 174.087(2.500) | 75.892(2.164) | 98.195(2.841) |  |  | 17(2.2) | 9(2.3) | 8(2.0) |  |  |
| ACEIs or ARBs, n (%) | 5196.294(74.622) | 2636.980(75.193) | 2559.314(74.043) | 0.715 | 0.026 | 536(68.0) | 272(69.0) | 264(67.0) | 0.593 | 0.044 |
| CCBs, n (%) | 1136.212(16.317) | 573.529(16.354) | 562.683(16.279) | 0.974 | 0.002 | 136(17.3) | 66(16.8) | 70(17.8) | 0.777 | 0.027 |
| Beta-blockers, n (%) | 5098.752(73.221) | 2574.886(73.422) | 2523.866(73.017) | 0.882 | 0.009 | 505(64.1) | 254(64.5) | 251(63.7) | 0.882 | 0.016 |
| Statins, n (%) | 6795.929(97.594) | 3410.899(97.261) | 3385.030(97.931) | 0.579 | 0.044 | 774(98.2) | 387(98.2) | 387(98.2) | 1.000 | <0.001 |
| Aspirin, n (%) | 6733.174(96.692) | 3377.780(96.317) | 3355.394(97.074) | 0.552 | 0.042 | 762(96.7) | 380(96.4) | 382(97.0) | 0.842 | 0.028 |
| Clopidogrel, n (%) | 5596.026(80.362) | 2777.145(79.190) | 2818.881(81.552) | 0.409 | 0.060 | 658(83.5) | 330(83.8) | 328(83.2) | 0.924 | 0.014 |
| Ticagrelor, n (%) | 1095.011(15.725) | 530.057(15.114) | 564.954(16.344) | 0.701 | 0.034 | 85(10.8) | 43(10.9) | 42(10.7) | 1.000 | 0.008 |
| Intravenous nitrates, n (%) | 4808.136(69.048) | 2391.550(68.194) | 2416.586(69.913) | 0.566 | 0.037 | 502(63.7) | 257(65.2) | 245(62.2) | 0.415 | 0.063 |
| Inotropes, n (%) | 1342.440(19.278) | 693.323(19.770) | 649.117(18.779) | 0.673 | 0.025 | 241(30.6) | 126(32.0) | 115(29.2) | 0.439 | 0.061 |

**Supplementary Table 1. (*continued*).**

| **Variables** | **IPTW adjustment ^1^** | | | | | **PSM adjustment ^2^** | | | | |
| --- | --- | --- | --- | --- | --- | --- | --- | --- | --- | --- |
|  | **All patients (N=6963.501)** | **Left ventricular infarction (N=3506.954)** | **Right ventricular infarction (N=3456.547)** | **P-value** | **SMD** | **All patients (N=788)** | **Left ventricular infarction (N=394)** | **Right ventricular infarction (N=394)** | **P-value** | **SMD** |
| Killip classification, n (%) |  |  |  | 0.561 | 0.105 |  |  |  | 0.864 | 0.061 |
| Ⅰ | 4625.849(66.430) | 2376.098(67.754) | 2249.751(65.087) |  |  | 548(69.5) | 271(68.8) | 277(70.3) |  |  |
| Ⅱ | 1621.751(23.289) | 767.825(21.894) | 853.926(24.705) |  |  | 141(17.9) | 73(18.5) | 68(17.3) |  |  |
| Ⅲ | 445.112(6.392) | 203.824(5.812) | 241.288(6.981) |  |  | 39(4.9) | 18(4.6) | 21(5.3) |  |  |
| Ⅳ | 270.790(3.889) | 159.207(4.540) | 111.582(3.228) |  |  | 60(7.6) | 32(8.1) | 28(7.1) |  |  |
| STEMI, n (%) | 4502.775(64.663) | 2360.491(67.309) | 2142.284(61.978) | 0.092 | 0.112 | 413(52.4) | 204(51.8) | 209(53.0) | 0.775 | 0.025 |
| NSTEMI | 2460.726(35.337) | 1146.464(32.691) | 1314.263(38.022) |  |  | 375(47.6) | 190(48.2) | 185(47.0) |  |  |
| HR, beats/min (median [IQR]) | 78(68-89) | 78(68-88) | 78(65-89) | 0.981 | 0.074 | 74(64-84) | 74(65-84) | 74(63-85) | 0.213 | 0.053 |
| SBP, mmHg (median [IQR]) | 130(115-146) | 130(115-145) | 131(113-149) | 0.239 | 0.081 | 125(110-141) | 125.00(110-142) | 125(110-140) | 0.755 | 0.013 |
| DBP, mmHg (median [IQR]) | 80(70-90) | 80(70-90) | 80(70-90) | 0.520 | 0.063 | 77(69-85) | 76(70-85) | 78(67-84) | 0.563 | 0.037 |
| FBG, mmol/L (median [IQR]) | 6.380(5.170-8.300) | 6.440(5.220-8.400) | 6.285(5.110-8.124) | 0.199 | 0.050 | 6.16(5.11-7.80) | 5.97(5.07-7.51) | 6.32(5.17-8.37) | 0.113 | 0.061 |
| LDL-C, mmol/L (median [IQR]) | 2.660(2.070-3.240) | 2.654(2.090-3.130) | 2.654(2.055-3.461) | 0.530 | 0.112 | 2.63(2.07-3.11) | 2.64(2.11-3.13) | 2.61(2.04-3.07) | 0.504 | 0.021 |
| LVEF, % (median [IQR]) | 54(45-60) | 53(45-60) | 54(45-60) | 0.353 | 0.010 | 55(49-62) | 54(48-62) | 56(50-61) | 0.757 | 0.001 |
| Medical history, n (%) | |  |  |  |  |  |  |  |  |  |
| Diabetes | 1388.245(19.936) | 685.362(19.543) | 702.883(20.335) | 0.780 | 0.020 | 141(17.9) | 70(17.8) | 71(18.0) | 1.000 | 0.007 |
| Hypertension | 3250.359(46.677) | 1642.805(46.844) | 1607.554(46.508) | 0.924 | 0.007 | 380(48.2) | 196(49.7) | 184(46.7) | 0.433 | 0.061 |
| Cardiac shock | 248.776(3.573) | 130.667(3.726) | 118.109(3.417) | 0.669 | 0.017 | 73(9.3) | 38(9.6) | 35(8.9) | 0.806 | 0.026 |
| Chronic heart failure | 184.509(2.650) | 76.368(2.178) | 108.141(3.129) | 0.369 | 0.059 | 25(3.2) | 16(4.1) | 9(2.3) | 0.223 | 0.101 |
| COPD or asthma | 323.479(4.645) | 160.251(4.570) | 163.228(4.722) | 0.904 | 0.007 | 45(5.7) | 22(5.6) | 23(5.8) | 1.000 | 0.011 |
| Stroke | 576.893(8.285) | 296.031(8.441) | 280.862(8.125) | 0.877 | 0.012 | 55(7.0) | 29(7.4) | 26(6.6) | 0.780 | 0.030 |
| Multivessel lesion, n (%) | 1170.636(16.811) | 552.862(15.765) | 617.774(17.873) | 0.354 | 0.056 | 181(23.0) | 89(22.6) | 92(23.4) | 0.865 | 0.018 |
| Revascularization, n (%) | 4642.229(66.665) | 2386.368(68.047) | 2255.861(65.263) | 0.422 | 0.059 | 562(71.3) | 276(70.1) | 286(72.6) | 0.478 | 0.056 |

**Abbreviation**: IPTW, inverse probability of treatment weighting; PSM, propensity score matching; SMD, standard mean difference; IQR, inter quartile range; BMI, body mass index; UEBMI, urban employee basic medical insurance; URBMI, urban resident basic medical insurance; NRCMS, new rural cooperative medical scheme; ACEIs or ARBs, angiotensin-converting enzyme inhibitors or angiotensin receptor blockers; CCBs, calcium channel blockers; STEMI, ST-elevation myocardial infarction; NSTEMI, Non-ST-segment elevation myocardial infarction; HR, heart rate; SBP, systolic blood pressure; DBP, diastolic blood pressure; FBG, fast blood glucose; LDL-C, serum low density lipoprotein-cholesterol; COPD, chronic obstructive pulmonary disease.

^1^ Adjusted for confounders including age, sex, health insurance, BMI, smoking status, in-hospital medications (ACEIs or ARBs, CCBs, beta-blockers, statins, aspirin, clopidogrel, intravenous nitrates and inotropes), medical history (diabetes, hypertension, stroke, chronic heart failure and COPD or asthma), STEMI or NSTEMI, cardiogenic shock, multivessel lesion, vital parameters and laboratory findings on admission (HR, SBP, DBP, FBG and LDL-C), LVEF, Killip classification and status of revascularization.

^2^ Matched for confounders including age, sex, health insurance, BMI, smoking status, in-hospital medications (ACEIs or ARBs, CCBs, beta-blockers, statins, aspirin, clopidogrel, intravenous nitrates and inotropes), medical history (diabetes, hypertension, stroke, chronic heart failure and COPD or asthma), STEMI or NSTEMI, cardiogenic shock, multivessel lesion, vital parameters and laboratory findings on admission (HR, SBP, DBP, FBG and LDL-C), LVEF, Killip classification and status of revascularization.
