# Supplementary material for: Clinical characteristics and prognosis differences between isolated right and left ventricular myocardial infarction in the Chinese population: a retrospective study
Source: PeerJ. 2023 Feb 28;11:e14959. doi: 10.7717/peerj.14959 (PMC9983429; doi:10.7717/peerj.14959)
Supplement: Supplemental Information 6 — Abbreviation: HR, hazard ratio. 1 Model 1 adjusted for confounders, including age, sex, health insurance, BMI, smoking status, in-hospital medications (ACEIs or ARBs, CCBs, beta-blockers, statins, aspirin, clopidogrel, intravenous nitrates and inotropes), medical history (diabetes, hypertension, stroke, chronic heart failure and COPD or asthma), STEMI or NSTEMI, cardiogenic shock, multivessel lesion, vital parameters and laboratory findings on admission (HR, SBP, DBP, FBG and LDL-C), LVEF, Killip classification and status of revascularization, by PSM method with a 1:1 ratio. 2 Model 2 additionally adjusted for confounders including laboratory findings on admission (Hb, WBC, peak cTnI, ALT, PLT, Cr and serum potassium), presenting symptoms (chest pain, chest stuffiness, shortness of breath and syncope), arrhythmia (atrial fibrillation, atrioventricular block, bundle branch block and bradycardia), echocardiography indices (LV and RV) besides abovementioned confounders. 3 Model 3 additionally IPTW-adjusted for confounders including laboratory findings on admission (Hb, WBC, peak cTnI, ALT, PLT, Cr and serum potassium), presenting symptoms (chest pain, chest stuffiness, shortness of breath and syncope), arrhythmia (atrial fibrillation, atrioventricular block, bundle branch block and bradycardia), echocardiography indices (LV and RV) besides abovementioned confounders. 4 Model 4 additionally adjusted confounders with SMD ≥0.05 after IPTW adjustment in model 3, which were age, health insurance, Hb, WBC, peak cTnI, PLT, Cr, serum potassium, LDL-C, echocardiography indices LV, chest stuffiness, STEMI or NSTEMI, bundle branch block, bradycardia and multivessel lesion. 5 model 5 used three hospitals as a random effect and adjusted for confounders, including age, sex, health insurance, BMI, smoking status, in-hospital medications (ACEIs or ARBs, CCBs, beta-blockers, statins, aspirin, clopidogrel, intravenous nitrates and inotropes), medical history (diabetes, hypertension, str [file peerj-11-14959-s006.docx]

**Supplementary Table 3. Adjusted HRs for left and right ventricular myocardial infarction in sensitivity analysis.**

| **Outcomes** | **Model 1 HRs^1^ (95%CI)** | **P-value** | **Model 2 HRs^2^ (95%CI)** | **P-value** |
| --- | --- | --- | --- | --- |
| All-cause mortality | 0.36(0.23-0.51) | <0.001 | 0.35(0.25-0.49) | <0.001 |
| Cardiovascular mortality | 0.30(0.18-0.51) | <0.001 | 0.31(0.19-0.48) | <0.001 |
|  | **Model 3 HRs^3^ (95%CI)** | **P-value** | **Model 4 HRs^4^ (95%CI)** | **P-value** |
| All-cause mortality | 0.39(0.26-0.61) | <0.001 | 0.38(0.25-0.58) | <0.001 |
| Cardiovascular mortality | 0.44(0.25-0.77) | 0.004 | 0.43(0.25-0.74) | 0.002 |
|  | **Model 5 HRs^5^ (95%CI)** | **P-value** |  |  |
| All-cause mortality | 0.35(0.25-0.49) | <0.001 |  |  |
| Cardiovascular mortality | 0.30(0.19-0.47) | <0.001 |  |  |

**Abbreviation**: HR, hazard ratio.

^1^ Model 1 adjusted for confounders, including age, sex, health insurance, BMI, smoking status, in-hospital medications (ACEIs or ARBs, CCBs, beta-blockers, statins, aspirin, clopidogrel, intravenous nitrates and inotropes), medical history (diabetes, hypertension, stroke, chronic heart failure and COPD or asthma), STEMI or NSTEMI, cardiogenic shock, multivessel lesion, vital parameters and laboratory findings on admission (HR, SBP, DBP, FBG and LDL-C), LVEF, Killip classification and status of revascularization, by PSM method with a 1:1 ratio.

^2^ Model 2 additionally adjusted for confounders including laboratory findings on admission (Hb, WBC, peak cTnI, ALT, PLT, Cr and serum potassium), presenting symptoms (chest pain, chest stuffiness, shortness of breath and syncope), arrhythmia (atrial fibrillation, atrioventricular block, bundle branch block and bradycardia), echocardiography indices (LV and RV) besides abovementioned confounders.

^3^ Model 3 additionally IPTW-adjusted for confounders including laboratory findings on admission (Hb, WBC, peak cTnI, ALT, PLT, Cr and serum potassium), presenting symptoms (chest pain, chest stuffiness, shortness of breath and syncope), arrhythmia (atrial fibrillation, atrioventricular block, bundle branch block and bradycardia), echocardiography indices (LV and RV) besides abovementioned confounders.

^4^ Model 4 additionally adjusted confounders with SMD ≥0.05 after IPTW adjustment in model 3, which were age, health insurance, Hb, WBC, peak cTnI, PLT, Cr, serum potassium, LDL-C, echocardiography indices LV, chest stuffiness, STEMI or NSTEMI, bundle branch block, bradycardia and multivessel lesion.

^5^ model 5 used three hospitals as a random effect and adjusted for confounders, including age, sex, health insurance, BMI, smoking status, in-hospital medications (ACEIs or ARBs, CCBs, beta-blockers, statins, aspirin, clopidogrel, intravenous nitrates and inotropes), medical history (diabetes, hypertension, stroke, chronic heart failure and COPD or asthma), STEMI or NSTEMI, cardiogenic shock, multivessel lesion, vital parameters and laboratory findings on admission (HR, SBP, DBP, FBG and LDL-C), LVEF, Killip classification and status of revascularization by performing the mixed effects COX model.
